# Assessment of symptom induction via artificial inoculation of the obligate biotrophic fungus *Phyllachora maydis* (Maubl.) on corn leaves

**DOI:** 10.1186/s13104-023-06341-y

**Published:** 2023-05-04

**Authors:** Carlos Góngora-Canul, Fidel E. Jiménez-Beitia, Carlos Puerto-Hernández, Mavir Carolina Avellaneda C., Nathan Kleczewski, Darcy E. P. Telenko, Sujoung Shim, José E. Solórzano, Stephen B. Goodwin, Steven R. Scofield, C. D. Cruz

**Affiliations:** 1 Department of Botany and Plant Pathology, Purdue University, 47907 West Lafayette, IN USA; 2 Tecnologico Nacional de México, Instituto Tecnológico de Conkal, 97345 Conkal, Yucatán Mexico; 3 Escuela Agrícola Panamericana Zamorano, Francisco Morazan, 93, San Antonio de Oriente, Honduras; 4 Department of Crop Science, University of Illinois, 61801 Urbana, IL USA; 5 Department of Plant Pathology, University of Minnesota, 55108 St. Paul, MN USA; 6 Crop Production and Pest Control Research Unit, USDA-Agricultural Research Service, 47907 West Lafayette, IN USA; 7 Department of Agronomy, Purdue University, West Lafayette, IN 47907 USA

**Keywords:** Corn, Induction, Maize, *Phyllachora maydis*, Signs, Stromata, Tar spot

## Abstract

**Objective:**

Tar spot is a foliar disease of corn caused by *Phyllachora maydis*, which produces signs in the form of stromata that bear conidia and ascospores. *Phyllachora maydis* cannot be cultured in media; therefore, the inoculum source for studying tar spot comprises leaves with stromata collected from naturally infected plants. Currently, there is no effective protocol to induce infection under controlled conditions. In this study, an inoculation method was assessed under greenhouse and growth chamber conditions to test whether stromata of *P. maydis* could be induced on corn leaves.

**Results:**

Experiments resulted in incubation periods ranging between 18 and 20 days and stromata development at the beginning of corn growth stage VT-R1 (silk). The induced stromata of *P. maydis* were confirmed by microscopy, PCR, or both. From thirteen experiments conducted, four (31%) resulted in the successful production of stromata. Statistical analyses indicate that if an experiment is conducted, there are equal chances of obtaining successful or unsuccessful infections. The information from this study will be valuable for developing more reliable *P. maydis* inoculation methods in the future.

**Supplementary Information:**

The online version contains supplementary material available at 10.1186/s13104-023-06341-y.

## Introduction

Tar spot of corn is an emerging disease caused by *Phyllachora maydis* Maubl. The disease is now distributed across the Americas [1]. In Mexico, the Caribbean, Central and South America, tar spot is considered to be caused by the interaction of three organisms: *P. maydis*, *Monographella maydis*, and *Coniothyrium phyllachorae* [2, 3]. However, *P. maydis* is the only pathogen linked with tar spot epidemics in the U.S. and Canada [4, 5].

*Phyllacora maydis* is proposed to be an obligate biotrophic fungus [6–9] because of its inability to obtain nutrients from dead cells and its dependence on living corn plants [10, 11]. However, recent studies suggest that *P. maydis* can survive in crop residue [12, 13].

Currently, no reliable and reproducible infection assay is available to study tar spot of corn. The isolation of *P. maydis* in synthetic media remains elusive, limiting the production of pure inoculum for pathological studies (1). However, infection assays using spores produced on plants exist for *Puccinia* and other fungi considered obligate biotrophs [14–16]. Conducting reliable inoculations that result in tar spot symptoms and signs under greenhouse or controlled conditions is critical. Such a method would allow the improved study of the biology and other aspects of the tar spot pathosystem. Therefore, this study aimed to test a *P. maydis* infection assay using spores produced on plants and evaluate the induction of corresponding symptoms and signs on corn leaves.

## Materials and methods

### Plant material and growth

In the greenhouse, seeds of the corn Hybrid P9998AM were sown in 3.05-L pots at a depth of 2.0 cm in a commercial soil mixture (Promix, Berber BM1™, Canada). Plants were fertilized with 200 ml/plot twice a week from growth stages V3 to V10 using the formula 24-8-16 (N-P-K; Miracle.Gro™, Marysville, OH), and from V11 to R2 (blister) growth stages with the formula 20-20-20. The planting date for the greenhouse experiment was 15 October 2018.

In the growth chamber, seeds of corn hybrids 2585-SS, Peaches & Cream, Honey Select Sweet Corn, and the inbred line B73 were used due to their susceptibility to the disease. Seeds were planted in plastic cones of 983 ml volume capacity at a 2.0-cm depth using a commercial soil mixture (Berger BM1™, Canada). Plastic cones were placed in plastic racks on plastic trays filled to one-third with water to ensure adequate plant growth. Plants were fertilized with 100 ml/cone twice a week from growth stages V3 to V10 using the formula 24-8-16 (Miracle.Gro™, Marysville, OH), and from the V11 to R2 growth stages using the formula 20-20-20. Experiments were planted on 10 June 2019.

### Inoculum preparation

**Fig. 1:**
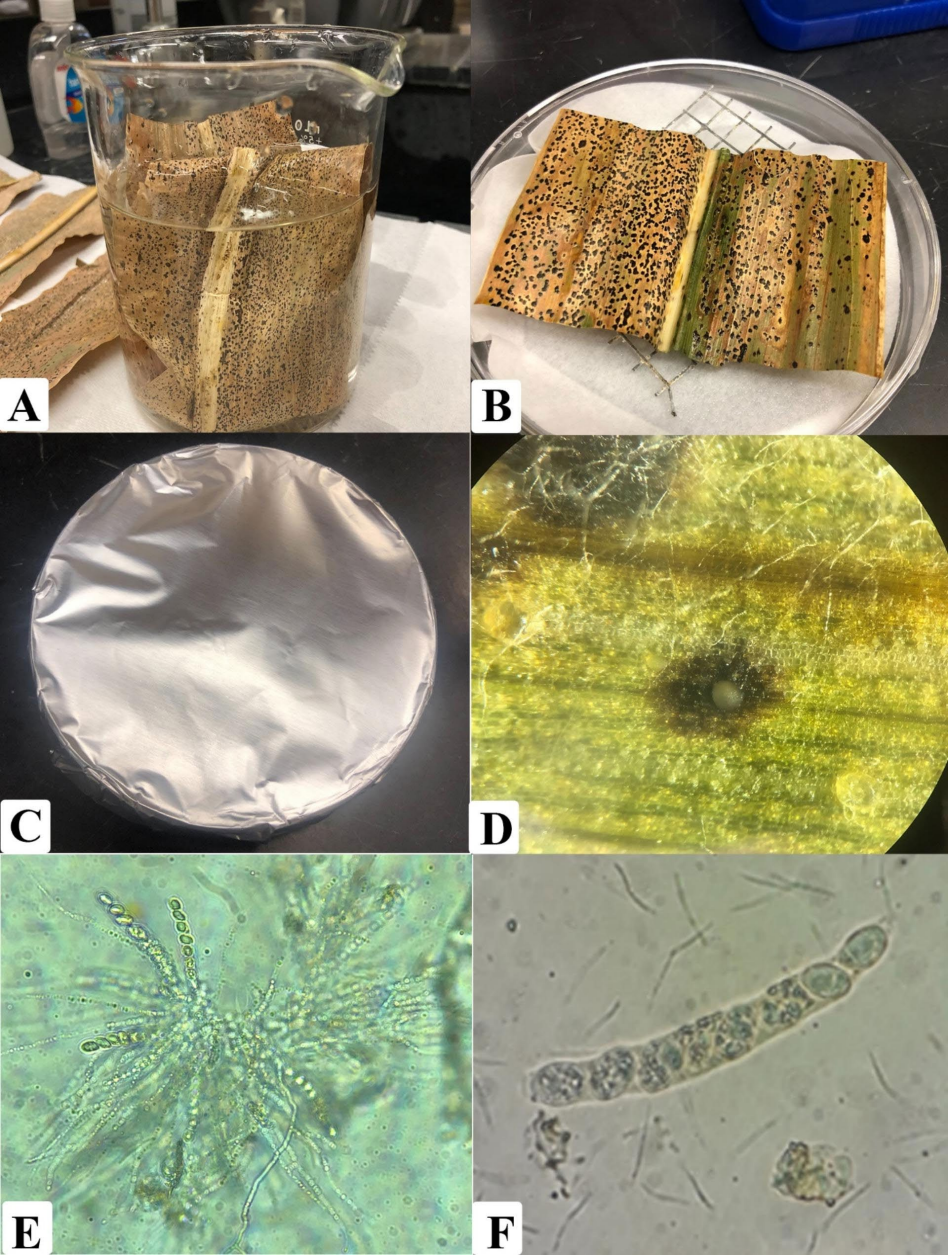
Inoculum preparation procedure. This included: **(A)** Surface sterilization of leaf samples. **(B)** Incubation of leaf samples inside a Petri plate. **(C)** Wrapping the plates with aluminum foil to ensure darkness. **(D)** Observation of a fruiting body with exudate under a dissecting microscope. **(E)** Observation of *P. maydis* ascospores in asci at 40x magnification. **(F)** Close-up of an ascus with eight ascospores

The inoculum was obtained from dried leaves with symptoms and signs of tar spot collected from fields in Indiana at the end of the corn production seasons of 2018 and 2019. Leaves were dried on a botanical press and stored in a refrigerator at 4 ^o^C. Before inoculation, slides of stromata were prepared and observed at 40x magnification using a compound microscope (Nikon ECLIPSE E100, Tokyo, Japan) to corroborate the presence of ascospores and conidia of *P. maydis*. Leaf samples were cut into approximately 3 × 3 cm pieces, surface sterilized with a solution of 10% sodium hypochlorite for 30 s, and rinsed twice with deionized sterilized water. Sterilized leaf samples were placed inside petri plates to create a humid chamber and incubated for 48 h at room temperature (23 °C). Plates were wrapped with aluminum foil to generate dark conditions. Then slides were prepared from the exudates of the stromata to corroborate the presence of *P. maydis* using a compound microscope at 40x magnification (Fig. 1).

### Inoculation assay

After confirming the presence of *P. maydis*, selected individual stromata were ruptured by teasing with a dissecting needle to release all available spores. The infection assay consisted of placing ruptured stromata with or without exudate in specific sections (infection court) of corresponding corn leaves. Inoculated leaves were marked in sections to track the development of the symptoms and signs over time. Two leaves per plant were inoculated. Leaf blades were divided into three infection court sections: (1) the base section was 1/3 of the leaf length beginning from the auricle; (2) the middle section was 1/3 of the length after the base section, and (3) the tip section was 1/3 of the leaf length towards the tip. Plants were inoculated at growth stages V9, VT, and R1.

### Incubation conditions

#### Greenhouse

Plants were inoculated on December 12 and 17, 2018, inside a greenhouse. Then plants were placed in a humid chamber for 72 h, with relative humidity (RH) > 95% (free water on leaves). The temperature was set at 25 °C day: 14 °C at night. The supplemental photoperiod consisted of 14 h of light (incandescent, 497 µMOL) and 10 h of dark (0.00 µMOL). Plants were watered as needed. Three days after inoculation, plants were taken out of the humid chamber and left in the greenhouse until stromata developed. The RH was 85%; temperature and photoperiod conditions were similar to those in the humid chamber. Supplementary tap water was sprayed onto the canopy of the plants twice daily (morning and afternoon) using a hose.

#### Growth chamber

Environmental conditions of the growth chamber were set at 85% RH using a misting system. Temperature and photoperiod were the same as described for the greenhouse experiments. Plants were watered as needed.

### Pathogen identification

If stromata formed, they were collected for identification purposes. Identification of *P. maydis* was conducted through microscopic observations of morphological characteristics for all experiments or by polymerase chain reaction (PCR) using the primers ITS1: 5’- TCC GTA GGT GAA CCT GCG G − 3’ and ITS4: 5’ - TCC TCC GCT TAT TGA TAT GC − 3’ [17].

### Experimental designs

A total of thirteen experiments were conducted, one under greenhouse, and twelve under growth chamber conditions. Experiments were conducted at Purdue University facilities in West Lafayette, Indiana, during 2018 and 2019. The design consisted of a completely randomized design with eight inoculated and two to four disease-free treated plants. The production of at least one stroma was considered a successful experiment, and each unit was scored as either successful or unsuccessful. This resulted in binary dataset (i.e., experiments with successful or unsuccessful infections), and SAS software (SAS, Cary, NC) was used for the analysis.

### Statistical analysis

Successful infection was recorded when at least a single stroma was formed and confirmed morphologically or by PCR. Due to the binary nature of the data, a single binomial test was used to compare the proportion of successful and unsuccessful infection experiments. A 95% confidence interval and a significance level of α = 0.05, were used. A Chi-square frequency test was also used. In both tests, the hypothesized proportion value ($\widehat{P}$) was 0.5 since successful and unsuccessful infection categories had an equal chance of occurring. Thus, for both tests, the null hypothesis and alternative hypothesis were:

H_o_: proportion of successful infections $\left(\widehat{P}\right)$ = hypothesized proportion$\left(P\right)$

H_a_: proportion of successful infections$\left(\widehat{P}\right)$ ≠ hypothesized proportion$\left(P\right)$

## Results

**Fig. 2:**
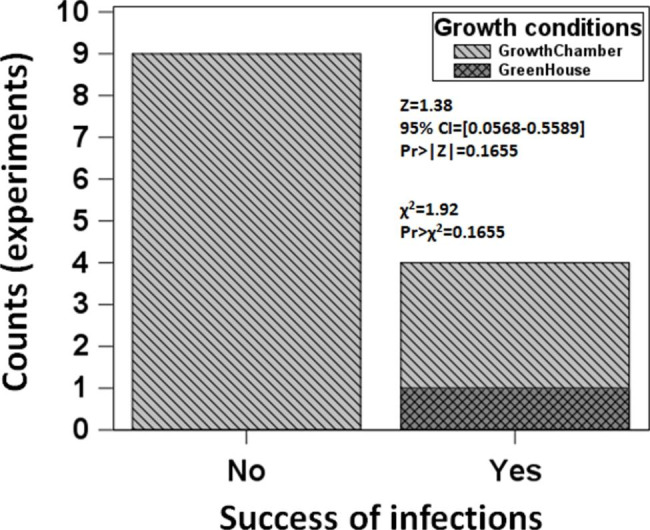
Proportion of experiments where stroma formation was achieved under greenhouse and growth chamber conditions. The x axis corresponds to unsuccessful (No) or successful (Yes) *P. maydis* infections. A total of 13 experiments was conducted, and an experiment was deemed successful after morphological and PCR confirmation when at least a single stroma was formed in a marked infection court

In total, successful *P. maydis* infection was achieved in four experiments out of thirteen conducted, one in the greenhouse and three out of the twelve experiments in the growth chamber (Fig. 2; Table 1). Multiple stromata formed per leaf on each successful count. Thus, the proportion of successful infection was p = 0.3077, and the proportion of non-success was q = 1-p = 0.6923 (Fig. 2). The binomial test failed to reject the null hypothesis (H_o_). The test statistics Z = 1.38 was less than the Z-critical value of 1.64. Then, the Pr>|Z|= 0.165 was higher than the significance level α = 0.05. Therefore, the proportion of successful infections p = 0.3077 was not different from the hypothesized proportion $\widehat{P }= 0.5$ and can occur at a proportion of 0.5. In other words, the infection assay proposed has an equal chance of getting successful or unsuccessful infections (Fig. 2).

**Table 1:** Successful development of *Phyllachora maydis* signs in inoculation experiments conducted under greenhouse and growth chamber conditions

Experiments #	#IP (#Str/P/#L) ^u^	I.P.^v^	AI^w^	GSI^x^	LPP^y^	LS^z^
**Greenhouse**						
1	2- (7/p/1L, 5 /p/1L)	20	2018	V9	4–6	Base
**Growth chamber**						
2	1- (5/p/1L)	21	2018	V11	5	Base
3	2- (1/p/1L, 2/p/2L)	21	2018	V11	7	Tip
4	1- (1/p/1L)	18	2019	VT-R1	9	Base

Note: The four experiments developed stromata. The type of spore used for inoculation was *P. maydis* ascospore, and the pathogen confirmed was *P. maydis* conidia using morphological and PCR techniques

^u^# IP-(#Str/P/#L) = number of infected plants (number of stromata per plant, and number of infected leaves)

^v^I.P. = incubation period in days

^w^AI= age of inoculum (year of collected inoculum)

^x^GSI=plant growth stage at inoculation

^y^LPP= leaf position in the plant (from lower leaf one to upper canopy)

^z^LS= leaf area section (base, middle and tip)

The Chi-square frequency test also failed to reject the null hypothesis (H_o_) since the χ^2^ = 1.92 is less than the critical value χ^2^_df=1_ = 3.84. Therefore, the proportion of successful infections p = 0.3077 was not different from the hypothesized proportion $\widehat{P} = 0.5$, or if an experiment is conducted, a successful or unsuccessful infection has the same chance of occurring (Fig. 2).

**Fig. 3:**
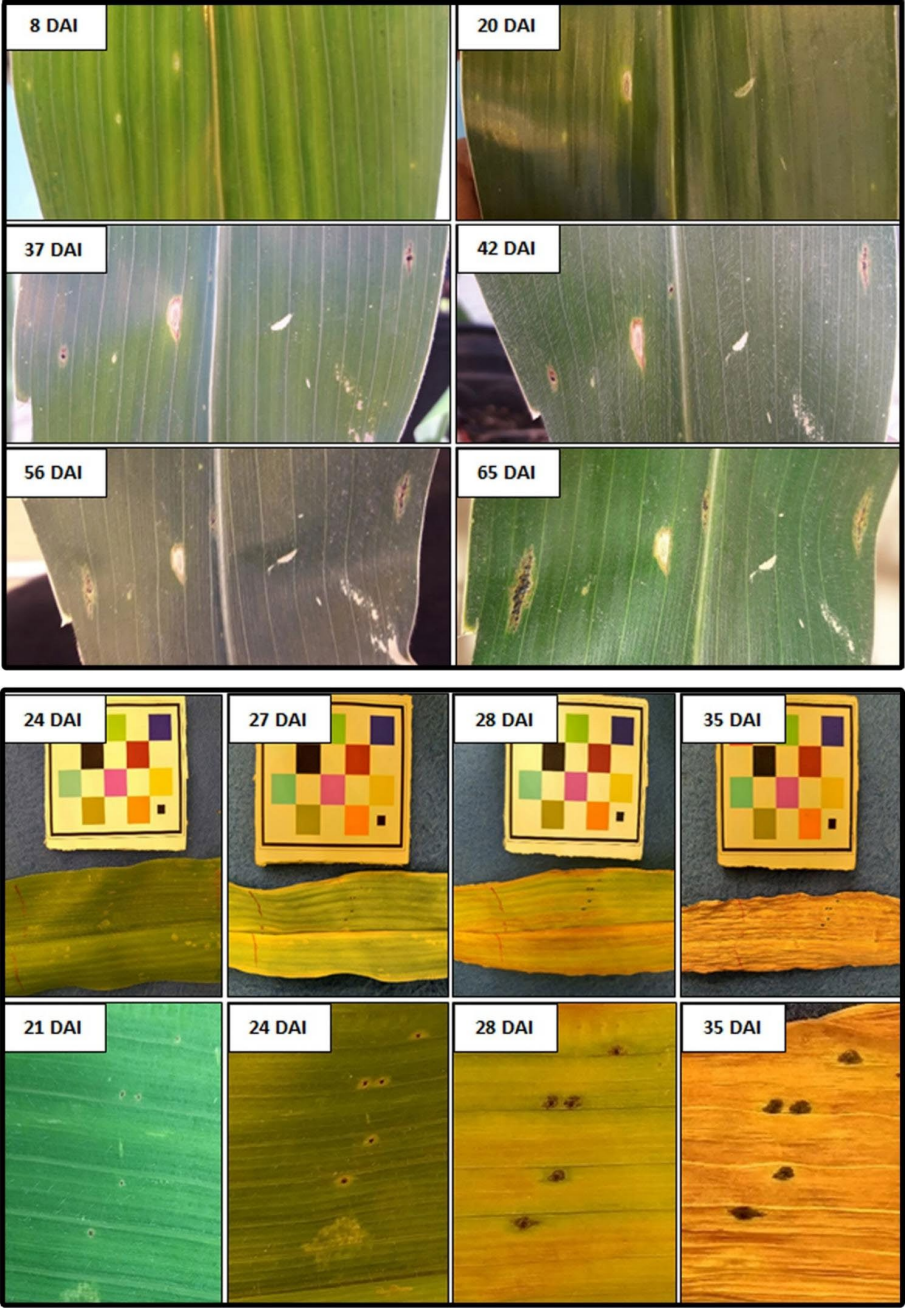
Progression of stromata formation several days after inoculation (DAI) on a corn leaf grown under greenhouse (upper) and growth chamber (lower) conditions

In the greenhouse, the infection assay provided the means for successful stromata development on two plants, with an incubation period of 20 days. Infection occurred on leaves 4–6 (from the lowermost leaf) and at the base section of each leaf. The symptoms began with small yellow lesions but were more elongated than those observed in growth chamber experiments. Gradually, small chlorotic lesions appeared 8 days after inoculation (DAI), then they became black with a regular and irregular shape with chlorotic borders at 37 DAI (Fig. 3). However, they did not have necrotic areas surrounding the stroma known as “fisheye lesions” under same conditions [18]. The stromata observed measured between 2 and 2.5 mm at 37 DAI and 4 to 5 mm long at 65 DAI (Fig. 3) (Table 1).

In the growth chamber, on each successful experiment, one or two plants developed multiple stromata. The incubation period ranged from 18 to 21 days. There was no clear pattern of infection regarding leaf position in the plant, leaf section, and genotype (Table 1). The symptoms began with small chlorotic lesions that gradually turned into black stromata with chlorotic borders. Some stromata had a regular oval shape, while others were irregular. The stromata measured between 0.5 and 1 mm at 21 DAI, and 2.5 to 3 mm long at 35 DAI (Fig. 3). Fisheye’s symptoms were not observed. The growth stage at inoculation was between V9 and VT, and symptoms were observed at the VT or R1 growth stages. No tar spot infections developed on any of the untreated leaves.

### Pathogen identification

**Fig. 4:**
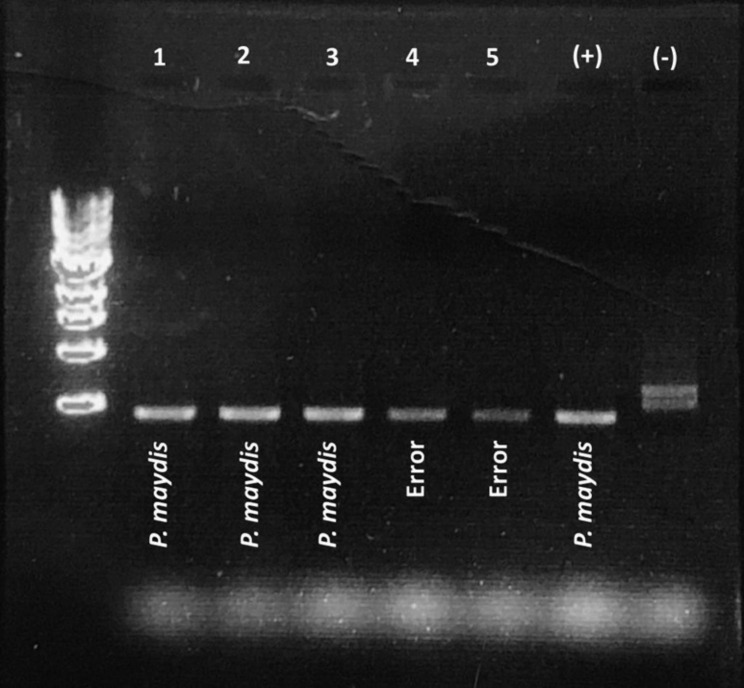
Molecular diagnosis of spores of *P. maydis* from stroma formed under greenhouse conditions

From all stromata formed under growth chamber and greenhouse conditions, only conidia of *P. maydis* were identified under the microscope at 40x. Molecular identification by PCR confirmed the identity of *P. maydis* in a limited number of samples with stromata collected from the greenhouse (Fig. 4).

## Discussion

Successful infections resulting in stromata formation were possible in 31% of all conducted experiments under controlled environments. Although we are one of the few groups in the U.S. that have reproducibly induced stroma formation post-inoculation, other studies have reported infections with low severity (1%) under controlled conditions [19]. However, our statistical results indicate a 50% chance of getting either successful or unsuccessful infections. Although results are limited, we report that the incubation period was between 18 and 20 days when inoculations occurred close to flowering at growth stages V9-VT, and symptoms were expressed at the VT-R1 (silk) growth stage. Since the scope of this study was to test the induction of *P. maydis* stromata, we could not determine the specific host-pathogen-environment interactions that allowed this to occur. Additionally, there was no clear pattern of stroma formation regarding leaf position, leaf section, and genotype. It is possible that inoculum age and quality might have influenced the germination capacity of spores used for inoculations [16, 20].

The function of conidia in the life cycle of *P. maydis* and their role in host infection are unclear [21]. Although limited, this is the first report of successful induction of *P. maydis* stromata on artificially inoculated plants in the U.S., becoming a valuable guide while we gain insights into the biology of *P. maydis* and its interaction with its host. Additional research is needed to identify underlying mechanisms that lead to the development of stromata as a result of host-pathogen-environment interactions. Future research about spore type, viability, age, and mechanism are required further to optimize an infection protocol [22, 23]. Additionally, it will be important to determine ranges of temperature, photoperiod, leaf wetness, and other weather variables during different times of the year. Finally, to understand if the growth stage of the host affects the formation of the spore germ tube and the general infection process of *P. maydis* [13, 20].

### Limitations

The study’s main limitation includes a 50% probability of obtaining a successful infection.

## Electronic supplementary material

Below is the link to the electronic supplementary material.

Supplementary Material 1

## Data Availability

Data has been made available as supplementary information.
